# Widely Tuneable Composition and Crystallinity of Graded Na_1+x_TaO_3±δ_ Thin Films Fabricated by Chemical Beam Vapor Deposition

**DOI:** 10.3390/nano12061012

**Published:** 2022-03-19

**Authors:** Corrado Garlisi, Petru Lunca Popa, Kevin Menguelti, Vincent Rogé, Marc Michel, Christèle Vergne, Jérôme Guillot, Estelle Wagner, William Maudez, Giacomo Benvenuti, Bianca Rita Pistillo, Emanuele Barborini

**Affiliations:** 1 Materials Research and Technology (MRT) Department, Luxembourg Institute of Science and Technology (LIST), L-4422 Belvaux, Luxembourg; petru.luncapopa@list.lu (P.L.P.); kevin.menguelti@list.lu (K.M.); vincent.roge@list.lu (V.R.); marc.michel@list.lu (M.M.); christele.vergne@list.lu (C.V.); jerome.guillot@list.lu (J.G.); biancarita.pistillo@list.lu (B.R.P.); emanuele.barborini@list.lu (E.B.); 2 3D-Oxides, F-01630 Saint Genis Pouilly, France; estelle.wagner@3d-oxides.com (E.W.); william.maudez@3d-oxides.com (W.M.); giacomo.benvenuti@3d-oxides.com (G.B.)

**Keywords:** chemical beam vapor deposition, compositionally graded Na_1+x_TaO_3±δ_, crystallinity spread, chemical reaction limited regime, sodium tantalate, perovskite

## Abstract

Combinatorial approach has been widely recognized as a powerful strategy to develop new-higher performance materials and shed the light on the stoichiometry-dependent properties of known systems. Herein, we take advantage of the unique features of chemical beam vapor deposition to fabricate compositionally graded Na_1+x_TaO_3±δ_ thin films with −0.6 < *x* < 0.5. Such a varied composition was enabled by the ability of the employed technique to deliver and combine an extensive range of precursors flows over the same deposition area. The film growth occurred in a complex process, where precursor absolute flows, flow ratios, and substrate temperature played a role. The deviation of the measured Na/Ta ratios from those predicted by flow simulations suggests that a chemical-reaction limited regime underlies the growth mechanism and highlights the importance of the Ta precursor in assisting the decomposition of the Na one. The crystallinity was observed to be strongly dependent on its stoichiometry. High under-stoichiometries (e.g., Na_0.5_TaO_3−δ_) compared to NaTaO_3_ were detrimental for the formation of a perovskite framework, owing to the excessive amount of sodium vacancies and oxygen vacancies. Conversely, a well-crystallized orthorhombic perovskite structure peculiar of NaTaO_3_ was observed from mildly under-stoichiometric (e.g., Na_0.9_TaO_3−δ_) to highly over-stoichiometric (e.g., Na_1.5_TaO_3+δ_) compositions.

## 1. Introduction

ABO_3_-type oxides with a perovskite structure have long served as a rich material playground for a plethora of applications due to their unique and multiple physicochemical properties such as superconductivity, ferroelectricity, ferromagnetism, catalytic activity, and ion conductivity [1,2,3,4]. Among ABO_3_ perovskite oxides, NaTaO_3_ has received much attention as an effective photocatalyst for water splitting and degradation of organic pollutants under ultraviolet radiation [5,6]. The piezoelectric properties of this material have also been investigated, showing a very promising potential for application in energy storage devices [7]. NaTaO_3_ consists of Na cations located at the corners of a pseudo-cubic unit cell and smaller Ta cations at the center of the cell and six-fold coordinated by oxygen, giving rise to corner-linked TaO_6_ octahedra [8]. NaTaO_3_ typically crystallizes in an orthorhombic unit cell while further crystal systems, such as tetragonal and cubic, closer to an ideal perovskite, can be observed at high temperatures (i.e., above 600 °C) [9,10].

A great research effort has been devoted to boosting the visible-light absorption of NaTaO_3_, achieved by the narrowing of its wide band gap (*E_g_* of ca. 4 eV) through the incorporation of dopants such as Bi, La, Fe, S, N, and C into the lattice [11,12,13,14]. In parallel to that, significant attention has been paid to further factors such as crystallinity, particle size, morphology, and presence of structural defects (e.g., oxygen vacancies), equally crucial for developing efficient photocatalysts. Such properties are synthesis route-dependent and have been effectively tuned by a wide range of wet chemical synthesis methods including hydrothermal [15,16], sol-gel [17], and coating Ta substrate with molten NaNO_3_, followed by annealing treatment [18,19]. However, few works report the fabrication of NaTaO_3_ thin films from vapor phase [20,21]. The only study dealing with Chemical Vapor Deposition (CVD) of NaTaO_3_ focuses on the effect of the process temperature (823–913 K) on the orientation and morphology of orthorhombic NaTaO_3_ [22]. In that work, the employed technique, laser beam CVD, made use of a high-power laser to promote the activation and reaction of the precursors on AlN substrates in Ar-O_2_ atmosphere.

Physicochemical properties of materials are primarily dependent on their composition. Indeed, a slight off-stoichiometry may, in principle, bring about different crystal phases and material properties, which, in turn, can dramatically impact on the functional performance. In this context, the development of non-stoichiometric perovskite oxides with associated point defects within the crystal lattice is increasingly becoming a hot topic in research. For example, an excess of bismuth can boost the piezoelectric properties of strontium bismuth tantalate [23]; in lithium tantalates, Li/Ta ratios diverging from the stoichiometric value, i.e., Li/Ta = 1, have been found to promote the ionic conductivity [24].

Chemical Beam Epitaxy (CBE) is a technique developed in the 90’s- to grow III-V semiconductors thin films, arising from the merging of CVD and MBE (Molecular Beam Epitaxy) [25]. Chemical Beam Vapor Deposition (CBVD), a variant of CBE with no epitaxy, relies on different molecular beams of chemical precursors effusing from an array of Knudsen-like sources into the deposition chamber under high vacuum conditions (10^−6^–10^−5^ mbar). As gas phase collisions are negligible, the precursor molecules reach the heated substrate with a line-of-sight pattern and thermally decompose. A multitude of combinatorial configurations are achievable with the possibility to obtain a wide range of flow compositions from the different precursors across the deposition area. In Sybilla equipment [26], the amount of each precursor reaching a given position of the substrate can, thus, be finely controlled and predicted by accurate calculations, enabling stoichiometry tuning. In addition to negligible gas phase collisions, CBVD has some further peculiarities compared to traditional CVD at higher pressure. CBVD does not require any carrier gas to deliver the precursor inside the deposition chamber. Given the nature of the precursor, that usually already contains oxygen, no reactive gases are normally required. Furthermore, decomposition is a pure surface mechanism with no pre-activation in the gas phase that can lead to powders and trapping of by-products in the boundary layer, with both having fewer chances to be incorporated into the thin film. The used precursor must, however, have a high sticking coefficient and enhanced decomposition probability on the surface to avoid molecules bouncing off the substrate without reacting [26].

The use of combinatorial approach with Sybilla equipment may open new horizons in fine-tuning properties of perovskites due to the possibility to access stoichiometries and phases not achievable by conventional solid-state methods while producing films with high growth rate, purity, and density.

In this work, we report, for the first time, the synthesis of compositionally graded Na_1+x_TaO_3±δ_ thin films obtained by CBVD in a Sybilla equipment, using different deposition temperatures and precursor flow rates, in two different source configurations. X-ray photoelectron spectroscopy (XPS) and time-of-flight secondary ion mass spectrometry (ToF-SIMS) were used to characterize the chemical composition, providing fundamental insight on the growth and marked off-stoichiometry of Na_1+x_TaO_3±δ_. The crystallinity and morphology were investigated, respectively, by X-ray diffraction (XRD) and Scanning Electron Microscopy (SEM)/Atomic Force Microscopy (AFM), and correlated to the composition of the deposited thin films.

## 2. Materials and Methods

### 2.1. Sybilla Equipment and Combinatorial Configurations

Sodium tert-butoxide (CAS 865-48-5, purity 98% min, Strem Chemicals, Newburyport, MA, USA,) and tantalum tetraethoxy dimethylaminoethoxide (CAS 172901-22-3, purity 98% min, EpiValence, Wilton, UK) were used as precursors for sodium and tantalum, respectively.

**Figure 1:**
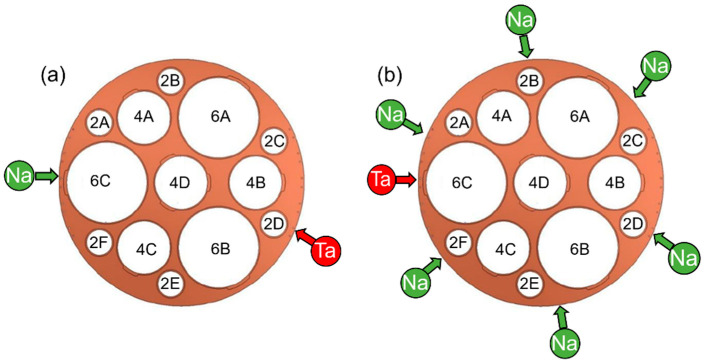
Schematics of the used deposition configurations: (**a**) 1-1 and (**b**) 1-6, where the red and green arrows show the angular positions of the active sources of tantalum and sodium, respectively. The sample holder (big circle, 450 mm) is able to accommodate 2” substrates (positions 2A, 2B, 2C, 2D, 2E, and 2F), 4” substrates (positions 4A, 4C, 4D, and 4B) and 6” substrates (positions 6C, 6B, and 6A).

The depositions were carried out in a CBVD system able to accommodate a 450 mm diameter substrate (Sybilla-450, ABCD Technology, Ferney-Voltaire, France). A similar equipment (Sybilla 150) and the operating mode of this technique are described elsewhere [26]. Substrate heating is achieved by a graphite heating system radiatively heating the substrate up to 800 °C. Silicon <100> and quartz wafers (Siegert Wafer, Aachen, Germany) were used as substrates. By using a sample holder able to accommodate substrates of various sizes (Figure 1), thin films were deposited on 2” (silicon, quartz), 4” (silicon), and 6” (silicon) wafers. Two deposition configurations were used: (i) one active source for both Ta and Na (1-1 configuration, Figure 1a); (ii) one active source for Ta and six for Na (1-6 configuration, Figure 1b).

In addition to results obtained on the samples located in 6C, 4D, and 4B, the data of 2A, 4C, 2B, 6A, and 2D will be reported for the 1-6 configuration. The latter samples are also representative of the samples 2F, 4A, 2E, 6B, and 2C due to the symmetric arrangement of the active sources. Similarly, the samples located in the same positions will be discussed for the 1-1 configuration despite the lack of symmetry, which may lead to slightly different compositions in 2F, 4A, 2E, 6B, and 2C compared to 2A, 4C, 2B, 6A, and 2D. The choice of the peculiar source configuration used in 1-1 deposition can be explained by the possibility of achieving a much higher range of compositions compared to 1-6 configuration. The two extreme stoichiometries are attained in positions 6C (closest to active Na source) and 2D (closest to active Ta source) and all the other samples are expected to exhibit an intermediate composition.

Precursors were evaporated at constant vapor pressure from thermostatically controlled reservoirs to the respective prechambers and then emitted through effusive sources. The lines connecting the precursors reservoirs to the prechambers were kept at a constant temperature throughout the process, i.e., 165 °C for Na precursor and 124 °C for Ta precursor. The effusive sources consisted of nine holes arranged in a 3 × 3 square pattern where the diameter of each hole was 1.5 mm. The vapor pressure was correlated to the reservoir temperatures by Arrhenius’ law. Different deposition sessions were carried out by changing the Na and Ta vapor pressures (VP) and deposition temperature (T_d_), as summarized in Table 1.

Flow rate calculations were performed similarly to a previous work [26]. The expected Ta and Na flows and the resulting flow ratios are estimated from precursor reservoir temperatures and target VP (Table 1), assuming that both precursors evaporate as monomers.

### 2.2. Characterization Techniques

The thickness of the samples was measured in a J.A. WOOLLAM M-2000 ellipsometer (J.A. Woollam Co., Lincoln, NE, USA) using a B-spline interpolation.

XPS was used to determine the film elemental composition using a Kratos Axis Ultra DLD spectrometer (Kratos Analytical Ltd, Manchester, UK) with a monochromatic Al K_α_ source operating at 105 W and an analysis area of 700 µm × 300 µm. Spectra were acquired with a pass energy of 80 eV to significantly increase the sensitivity of the detector and the scanning speed, thus allowing the characterization of numerous samples while quantifying accurately the elements in low concentration. The peak fit models for carbonates quantification and tantalum chemical states identification were determined on spectra acquired with a pass energy of 20 eV and then applied to 80 eV pass energy spectra (peaks positions were fixed, slightly broader full-width-at-half-maxima were allowed). XPS spectra were acquired before and after a mild etching (500 V-Ar^+^-t = 60 s) of the surface to remove most of the possible surface contaminants deriving from reaction with the air of the topmost material layers. The reported elemental compositions are those calculated from the narrow scans obtained after the mild etching.

ToF-SIMS using an IonTOF TOFSIMS 5 apparatus (IONTOF, Münster, Germany) was carried out to further characterize the surface composition. A pulsed beam of bismuth primary ions (Bi_3_^+^) with a dose of 1e^+11^ ions/cm^2^ (pulsed current 0.31 pA) was employed to sputter the samples.

XRD measurements were performed with a BRUKER D8 Discover Series 2 diffractometer (Billerica, MA, USA) using CuK_α_ as a radiation source. Diffraction patterns were recorded in the range of diffraction angles 2θ from 20° to 70° with a grazing angle of 0.5°, step size of 0.02° and scan speed of 2°/s. The diffractometer operated in parallel beam configuration with 1.2 mm solar slit and 1.2 mm collimator; the distance of the sample from the collimator was 40 cm. The degree of crystallinity (DOC) method was used to estimate the percentage of crystalline material. According to this method, the DOC is calculated by dividing the total area of crystalline peaks by the total area under crystalline and amorphous components [27,28].

The morphology of the deposited films was studied by SEM with a FEI HELIOS NanoLab microscope (FEI, Hillsboro, OR, USA). To estimate the surface roughness (root mean square roughness, *R_q_*), further morphological investigation of the samples surface was performed by AFM with an OXFORD MFP-3D Infinity microscope (Oxford Instruments, Abingdon-on-Thames, UK).

Optical properties of the samples deposited on quartz were studied in the range 250–2500 nm by UV/Vis/NIR spectroscopy on a PerkinElmer LAMBDA 1050 spectrometer (Waltham, MA, USA). The optical band gap was obtained by the Tauc’s relation for direct allowed transitions:

(1) $$\alpha h\nu =A{\left(h\nu -E_{g}\right)}^{1/2}$$

where *A* is a constant, *ν* is the frequency of the incident radiation, *h* is the Planck constant, and *E_g_* is the optical band gap.

## 3. Results and Discussion

### 3.1. Growth Process

**Figure 2:**
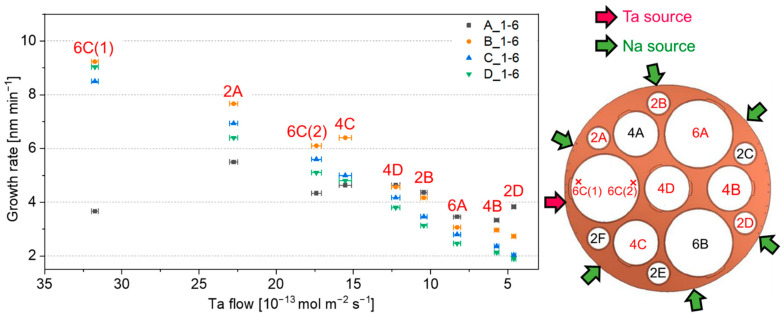
Measured average growth rate as function of the Ta flow calculated for each position of the samples in 1-6 batches. On the right, the schematic of the sample holder with the corresponding positions in red. The samples 6C(1) and 6C(2) come from the left edge and right edge, respectively, of the wafer 6C. The other samples come from the center of the respective wafers. Error bars refer to the Ta flows calculated at ±5 mm away from the point where thickness measurements were performed, thus considering possible measurement errors related to the positioning of the sample.

Chemical reaction limited regime (CRLR) and mass transport limited regimes (MTLR) are the two main growth regimes in a CBVD process and the occurrence of one or the other depends mainly on substrate temperature and precursor flow [29]. The study of the film compositions and the growth rates enables to determine the working regime. Table S1 reports the average thickness along with the corresponding standard deviation. The measured average growth rate in 1-6 batches as a function of the calculated Ta flow for various sample positions is reported in Figure 2. The same plot for 1-1 deposition is shown in Figure S1. The deposition run performed in the latter configuration exhibited growth rates up to eighteen times lower compared to those obtained in 1-6 configuration due to the lower precursors flows. The growth rates were similar in C_1-6 and D_1-6 performed at different temperature with the same precursor flows. The higher Na precursor flow in B_1-6 resulted only in a minor increase in the growth rate, suggesting a CRLR with Ta being the limiting element in the mechanism. Lower growth rates were found in A_1-6 in the presence of high Ta flow due to the lower deposition temperature (480 °C) and thus to a more pronounced CRLR. However, the trend was reversed at lower Ta flow. In CRLR, the reaction kinetics determines the film growth, which is affected simultaneously by multiple factors including reaction mechanism, substrate temperature, and absolute flow values, as well as precursor flow ratios. In this regime, not all the precursor molecules are incorporated into the film due to the low decomposition kinetics.

**Figure 3:**
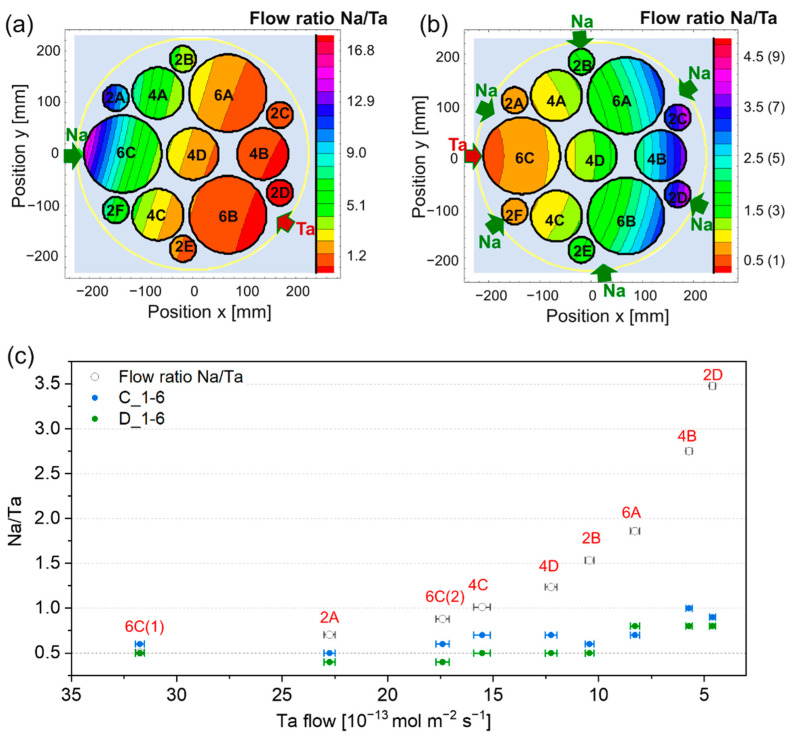
Flow simulations for (**a**) 1-1 and (**b**) 1-6 batches. In the latter case, scale values for Na/Ta flow ratios are for depositions C and D (target VP_Na_= 0.01 mbar), while values in brackets are for depositions A and B (target VP_Na_= 0.02 mbar). (**c**) Comparison between the measured Na/Ta by XPS and Na/Ta calculated from flow simulations in batches C_1-6 and D_1-6. For the position of the samples and active sources, refer to Figure 2. Error bars refer to the Ta flows calculated at ±5 mm away from the point where thickness measurements were performed, thus considering possible measurement errors related to the positioning of the sample.

The results rule out a possible transition from CRLR to a MTRL, usually occurring with increasing temperature. Unlike CRLR, in MTLR, the deposited thickness is directly proportional to the precursor flow since all precursor molecules reaching the substrate decompose and are ideally incorporated in the film. The results of the flow simulations obtained under this regime are show in Figure 3a,b for 1-1 and 1-6 configurations, respectively. Predictably, the 1-1 configuration led to a wider range of flow ratios due to the peculiar arrangement of the active sources (Figure 1a). The Na/Ta ratios measured in the deposited thin films by XPS were significantly lower than those predicted by flow simulations, as depicted in Figure 3c for depositions C_1-6 and D_1-6. This confirms that not all Na atoms in the precursors flow are incorporated in the material and thus that the process occurs in CRLR. Moreover, the same regime is also present at higher temperatures (620 °C in D_1-6), indicating that the increase in temperature does not allow to achieve MTLR. Finally, at low Ta flows, the amount of Na not contributing to the film growth increases. This is confirmed by the greater divergence between measured and calculated Na/Ta with decreasing Ta flow. A sufficiently high Ta flow (e.g., > ~25 × 10^−13^ mol m^−2^ s^−1^ in C_1-6 and D_1-6) is thus needed to ensure the decomposition of Na precursor and fully incorporate the impinging Na molecules in the growing film.

### 3.2. Chemical Composition

The stoichiometry of the films determined by XPS is reported in Table S2. The wide range of compositions of Na_1+x_TaO_3±δ_ spanned from under-stoichiometric to over-stoichiometric NaTaO_3_ with −0.6 < *x* < 0.5. The Ta 4f spectrum was best fitted with a doublet at binding energies (BE) ~27.5 eV (Ta 4f_5/2_) and ~25.7 eV (Ta 4f_7/2_), while the Na 1s and O 1s peaks (data not shown) were located at ~1071.2 and 529.9 eV, respectively. These values are consistent with the position of NaTaO_3_ reported in literature [30,31]. The analysis of the C 1s core level spectrum revealed an increase in the CO_3_^2−^ content with increasing Na/Ta ratio. In this respect, Figure S2 displays the trend in the amount of carbonate species as a function of the sample position and the corresponding Na/Ta ratio for the batch 1_1. The formation of surface CO_3_^2−^ is in line with other studies reporting the same findings on similar perovskite-like materials, such as LiNbO_3_ and LaFeO_3_ [32,33]. Such species mainly originate following air exposure of the sample surface.

**Figure 4:**
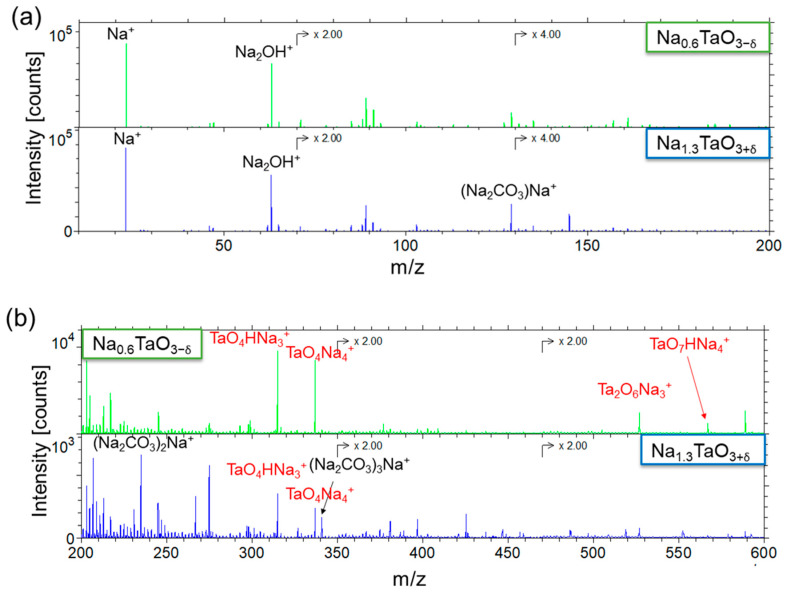
Positive ion ToF-SIMS spectra of Na_0.6_TaO_3-δ_ and Na_1.3_TaO_3+δ_ in the m/z range (**a**) 100–200 and (**b**) 200–600. Both samples were obtained from batch 1_1 (positions 4B and 6C(1), respectively). Only signals ascribed to Na_1+x_TaO_3±δ_ and Na_2_CO_3_ are reported. The secondary ions characteristic of Na_1+x_TaO_3±δ_ are labelled in red.

ToF-SIMS was performed to elucidate the contribution of the surface species to the chemical composition. Figure 4 shows the positive ion ToF-SIMS spectra of Na_1.3_TaO_3+δ_ and Na_0.6_TaO_3−δ_ deposited in batch 1-1. Both samples generate TaO_4_HNa_3_^+^, TaO_4_Na_4_^+^, Ta_2_O_6_Na_3_^+^, TaO_7_HNa_4_^+^, and TaO_7_Na_5_^+^, which confirm the presence of Na_1+x_TaO_3±δ_. On the other hand, (Na_2_CO_3_)_y_Na^+^ (with y = 1–3) ions, characteristic of Na_2_CO_3_, are emitted only from Na_1.3_TaO_3+δ_. No secondary ions distinctive of Ta_2_(CO_3_)_5_ were detected, implying that only surface Na_2_CO_3_ was formed. Albeit in a lower amount with respect to the thin film surface, CO_3_^2−^ was also spotted in the bulk as revealed by XPS depth profile analysis (not shown here). This suggests that, under Na-excess conditions, carbonates species originate from both the growth process and, to a larger extent, following a reaction, upon air exposure, between CO_2_ and residual unreacted Na precursor adsorbed on the surface at the end of the process.

**Figure 5:**
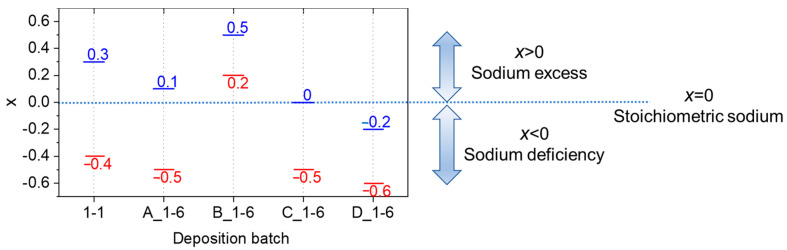
Maximum (in blue) and minimum (in red) values of *x* for each deposition batch.

The minimum and maximum values of *x* and *δ* achieved in each deposition batch (see Table 1 for deposition parameters) are displayed in Figure 5. The batch 1-1 showed the widest composition range with −0.4 ≤ *x* ≤ 0.3 around the stoichiometric composition attained in 6A position. The higher compositional spread compared to 1-6 configuration was enabled by the larger range of precursor flow ratios across the deposition area as discussed in Section 3.1. Stoichiometric NaTaO_3_ was also deposited in batch C_1-6, mostly characterized by under-stochiometric oxides (−0.5 ≤ *x* ≤ 0.0) with Na and O depletion. Slightly lower *x* values were found in batch D_1-6 (−0.6 ≤ *x* ≤ −0.2) deposited at the highest deposition temperature with same precursors flows as those in C_1-6. The lower *x* in D_1-6 may be ascribed to a preferential volatilization of Na in the growing perovskite structure at higher temperature, resulting in more Na vacancies [34].

A wide composition range was also obtained in the batch A_1_6 (−0.4 ≤ *x* ≤ 0.3), deposited at the lowest temperature and highest Na flow. Here, all the samples were characterized by oxygen deficiency (see Table S2) even in presence of sodium excess (i.e., *x* > 0). This charge unbalance can be due to the lack of a perovskite-structured material with possible deviations from the proposed A_1+x_BO_3±δ_ stoichiometry, as discussed in Section 3.3. Finally, batch B_1-6 presented over-stoichiometric oxide (0.2 ≤ *x* ≤ 0.5) across the whole deposition area.

Under sodium-rich conditions, replacement of Ta^5+^ by Na^+^ ions is highly unlikely due to: (i) the large difference between the charge state and the ionic radius, being 1.39 Å for Na^+^ and 0.64 Å for Ta^5+^ [35], (ii) much lower electronegativity of Na^+^, i.e., 0.956, compared to that of Ta^5+^, i.e., 1.881 [36,37]. It can, therefore, be inferred that the excess of Na in Na_1+x_TaO_3+δ_ (*x* ≥ 1) is incorporated interstitially. Similarly, the corresponding extra oxygen is reported to occupy interstitial positions [38]. The above-mentioned proliferation of carbonate species at increasing high Na/Ta flow ratios is emblematic of the reduced proneness to host Na^+^ within the lattice of over-stoichiometric oxides. Indeed, the too close-packed perovskite structure hinders more and more the incorporation of Na-excess which is increasingly consumed as sodium carbonate. The sample Na_1.5_TaO_3+δ_ obtained in the batch B_1-6 was the one with the largest over-stoichiometry, suggesting that Na_1+x_TaO_3+δ_ cannot accommodate additional Na^+^ above Na/Ta > 1.5, i.e., *x >* 0.5, and a further increase in the Na flow would only result in the formation of Na_2_CO_3_ and larger amount of unreacted Na precursor. These observations are consistent with the flow simulation results which revealed the occurrence of a CRLR and a precursors flow with an excess of Na not incorporated in the material (Section 3.1).

### 3.3. Cristallinity

**Figure 6:**
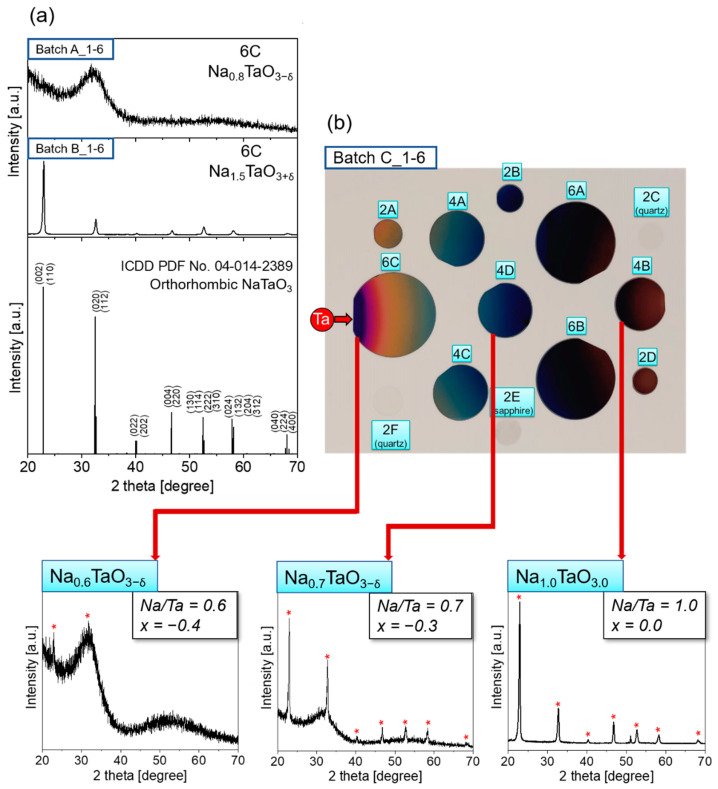
(**a**) XRD patterns of the sample located in 6C position in batches A_1-6 and B_1-6. (**b**) Photographic picture of the batch C_1-6 with the XRD patterns of the samples 6C, 4D, and 4B.

Figure 6a displays the XRD patterns of two samples deposited in 1-6 configuration at different temperatures with the same precursor flows. An amorphous phase was obtained at T_s_ = 480 °C, while increasing T_s_ to 550 °C promoted the growth of orthorhombic structure (ICDD PDF Card No: 04-014-2389, orthorhombic NaTaO_3_) as shown for the samples in position 6C. No impurities of other phases were detected. Notably, the whole batch B_1-6 was highly crystalline, while batch A_1-6 was fully amorphous.

Single phase orthorhombic structure was also deposited at T_d_ ≥ 550 °C when decreasing the flow of Na as done in C_1-6 and D_1-6. However, in these two batches, the crystallinity was strongly dependent on the precursors flow ratio as confirmed by the gradual appearance of orthorhombic lattice with increasing Na/Ta. Figure 6b shows a photographic picture of the batch C_1-6 with the XRD patterns of the samples deposited along the Ta gradient direction (6C, 4D and 4B). Moving away from the Ta source, the increase in Na/Ta ratio is accompanied by a rise in the crystallinity: Na_1+x_TaO_3±δ_ switches from a barely crystalline structure at Na/Ta = 0.6 (i.e., Na_0.6_TaO_3-δ_), to a well-crystallized orthorhombic phase at Na/Ta = 1.0 (i.e., Na_1.0_TaO_3.0_), passing through an intermediate crystallinity at Na/Ta = 0.7 (i.e., Na_0.7_TaO_3-δ_). In other words, the increasing precursor flow ratio along the Ta gradient involved a transition from under-stoichiometric to stoichiometric composition with an improvement in the crystalline order.

**Figure 7:**
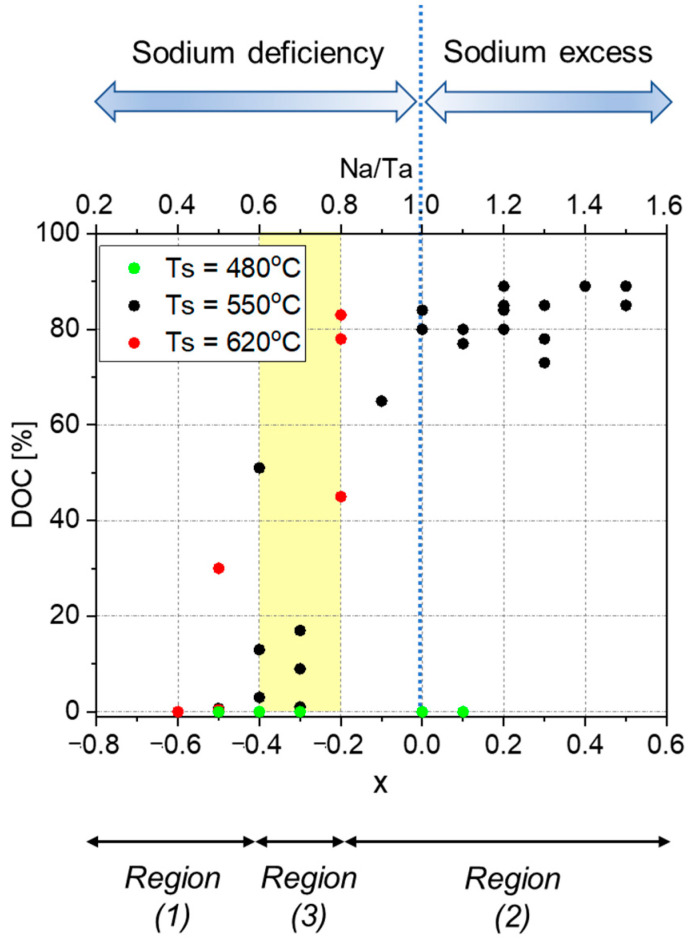
DOC versus *x* in Na_1+x_TaO_3±δ_ (bottom x-axis) and Na/Ta (top x-axis). Data include all the samples deposited in the deposition sessions summarized in Table 1, for a total of forty points analyzed. The area in yellow represents the transition region.

The tight correlation between stoichiometry and crystallinity, with the latter expressed in terms of the DOC, is highlighted in Figure 7, where a total of forty points across the deposition area, from all the deposition sessions of the present study, have been analyzed. Amorphous structure was observed at T_s_ = 480 °C at any stoichiometry. On the other hand, at temperatures of 520–620 °C, high enough to trigger the growth of perovskite-structured Na_1+x_TaO_3±δ,_ the crystalline order depended solely on the composition. Indeed, three main regions stand out:

(1) at *x* < −0.4 (i.e., Na/Ta < 0.6), Na_1+x_TaO_3-δ_ is mostly amorphous/nanocrystalline with DOC in the range 0–30%;

(2) at *x* > −0.2 (i.e., Na/Ta > 0.8), the material is characterized by a well-crystallized orthorhombic structure (DOC above 65%);

(3) at −0.4 ≤ *x* ≤ −0.2 (i.e., 0.6 ≤ Na/Ta ≤ 0.8), the wide crystallinity spread (DOC in the range 0–82%) suggests the occurrence of a threshold region.

Such a trend indicates a breakdown in the perovskite framework at extreme under-stoichiometries owing to the excessive Na and O deficiency. High crystallinity is instead observed from mild under-stoichiometries to marked over-stoichiometries. It can, thus, be concluded that the excess of Na and O located at interstitial sites, do not disrupt the crystal quality as much as their high deficiency in the cationic and anionic sites, respectively, of Na_1+x_TaO_3±δ_ lattice. Interestingly, the crystal growth at 620 °C is promoted at a slightly lower under-stoichiometry as confirmed by the sample Na_0.5_TaO_3−δ_ (batch D_1-6, position 2B) with DOC = 30%. The higher deposition temperature is, therefore, able to partly compensate for the structural disorder caused by anionic and cationic vacancies in under-stoichiometric Na_1+x_TaO_3−δ_.

### 3.4. Morphological and Optical Properties

**Figure 8:**
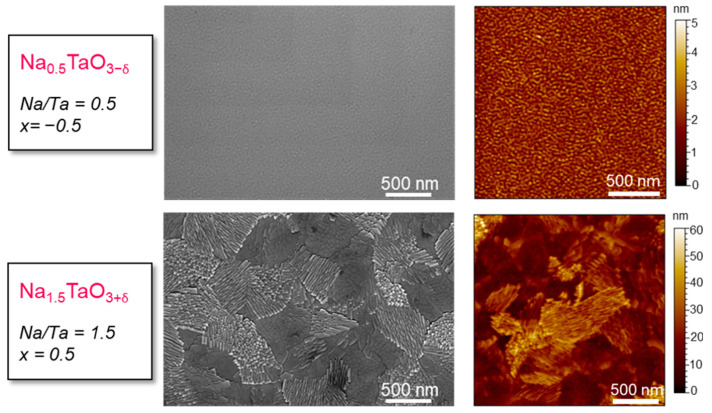
SEM (**left**) and AFM (**right**) images of Na_0.5_TaO_3−δ_ and Na_1.5_TaO_3+δ_. The morphology confirms high crystallinity and the amorphous character of Na_1.5_TaO_3+δ_ and Na_0.5_TaO_3−δ_, respectively.

Figure 8 shows SEM and AFM pictures of Na_0.5_TaO_3-δ_ and Na_1.5_TaO_3+δ_ deposited in position 6C from D_1-6 and B_1-6 batches, respectively. The different structural properties between the two oxides translated into a dissimilar morphology. The under-stoichiometric oxides did not present any significant surface texture, showing a relatively smooth surface as confirmed by the low roughness (*R_q_* = 3.5 nm in Na_0.5_TaO_3−δ_). The film structure evolved to a morphology typical of polycrystalline materials as Na/Ta increased. Indeed, the morphologies of stoichiometric and over-stoichiometric samples showed well-defined crystalline grains with an enhanced roughness (*R_q_* = 7.2 nm in Na_1.5_TaO_3+δ_). It is worth highlighting that the samples have a similar thickness, hence the presence of any growth dynamics causing thickness-related surface morphology can be definitely ruled out.

Optical characterization revealed a band gap of ca. 4.8 eV at any stoichiometry, larger than that reported for bulk NaTaO_3_ [11]. Figure S3 displays the Tauc plots of Na_0.4_TaO_3−δ_ and Na_1.2_TaO_3+δ_ deposited on quartz in position 2A from D_1-6 and B_1-6 batches, respectively. Band gap-tuning can effectively occur when dealing with atoms with comparable size, an important prerequisite for substitutional replacement to take place. In our case, the absence of substantial change in E_g_ with increasing Na/Ta ratio arises from the large difference in charge and ionic size between the involved cation species, which precludes the substitution of Ta^5+^ by Na^+^ within the lattice of Na_1+x_TaO_3±δ_. A similar result has been reported for halide perovskites [39].

## 4. Conclusions

Na_1+x_TaO_3±δ_ thin films with a wide range of compositions, spanning from Na_0.4_TaO_3-δ_ to Na_1.5_TaO_3+δ_, were fabricated for the first time by CBVD. The difference between the calculated and measured Na/Ta, especially at low Ta flow, was indicative of a growth process taking place in CRLR and suggested that a large excess of Na is not incorporated in Na_1+x_TaO_3±δ._ Interestingly, the decomposition of Na precursor and its inclusion in the final material were triggered by the Ta precursor since the excess of unreacted Na was significantly reduced at high Ta flow.

The occurrence of a perovskite-type compound was linked to the deposition temperature and composition of the deposited materials. Low deposition temperature (480 °C), as well as high under-stoichiometry (Na/Ta < 0.6), led to the growth of amorphous phase, while a highly crystalline orthorhombic structure was observed at 550 °C and 620 °C from moderate under-stoichiometries (Na/Ta > 0.8) to high-over stoichiometries (Na/Ta = 1.5). A transition region with large crystallinity spreads occurred at intermediate Na/Ta (0.6–0.8). The reason behind the gradual deterioration of the crystal quality in highly under-stoichiometric oxides was the excessive deficiency of A-sites and O-sites which prevented the growth of the perovskite network. On the other hand, the extra Na incorporated interstitially into the Na_1+x_TaO_3±δ_ lattice at high Na flow did not undermine the crystal growth despite the enhanced formation of carbonates caused by a large amount of unreacted Na precursor.

The combinatorial investigation of Na_1+x_TaO_3±δ_ presented in this work can have a significant impact on design strategies and future application of sodium tantalate-based thin films, such as in photocatalysis and piezoelectric materials, with the possibility of developing increasingly efficient materials having widely tuneable stoichiometry and crystal structure. The employed deposition technique can play a primary role in this scenario due to its unique power of unravelling previously unexplored composition and phases.

## Figures and Tables

**Table 1:** List of the deposition sessions.

Deposition Batch	Configuration	Target VP_Ta_^1^ (mbar)	Target VP_Na_^1^ (mbar)	T_d_ (°C)
1-1	1-1	0.02	0.02	550
A_1-6	1-6	0.1	0.02	480
B_1-6	1-6	0.1	0.02	550
C_1-6	1-6	0.1	0.01	550
D_1-6	1-6	0.1	0.01	620

^1^ The measured values showed fluctuations leading in some cases to slight deviations, i.e., up to ±20%, from the target values.

## Data Availability

Not applicable.

## Supplementary Materials

The following supporting information can be downloaded at: https://www.mdpi.com/article/10.3390/nano12061012/s1. Figure S1: Measured average growth rate as function of the Ta flow calculated for each position of the samples for the 1-1 deposition batch; Figure S2: Carbonate amount versus sample position for the batch 1-1; Figure S3: Tauc plots of the samples Na_0.4_TaO_3−δ_ and Na_1.2_TaO_3+δ_; Table S1: Average thickness with the corresponding standard deviation; and Table S2: Stoichiometry, Na/Ta and *x* of samples deposited in the five deposition batches.
