# Supplementary material for: Widely Tuneable Composition and Crystallinity of Graded Na1+xTaO3±δ Thin Films Fabricated by Chemical Beam Vapor Deposition
Source: Nanomaterials (Basel). 2022 Mar 19;12(6):1012. doi: 10.3390/nano12061012 (PMC8948640; doi:10.3390/nano12061012)
Supplement: Supplementary file 1 [file nanomaterials-12-01012-s001.zip › nanomaterials-1633013-supplementary.pdf]

Supplementary Materials

Widely Tunable Composition and Crystallinity of Graded $Na_{1+x}TaO_{3\pm\delta}$ Thin Films Fabricated by Chemical Beam Vapor Deposition

Corrado Garlisi [1,*], Petru Lunca Popa [1], Kevin Menguelti [1], Vincent Rogé [1], Marc Michel [1], Christèle Vergne [1], Jérôme Guillot [1], Estelle Wagner [2], William Maudez [2], Giacomo Benvenuti [2], Bianca Rita Pistillo [1] and Emanuele Barborini [1]

[1] Materials Research and Technology (MRT) Department, Luxembourg Institute of Science and Technology (LIST), L-4422 Belvaux, Luxembourg; petru.luncapopa@list.lu (P.L.P.); kevin.menguelti@list.lu (K.M.); vincent.roge@list.lu (V.R.); marc.michel@list.lu (M.M.); christele.vergne@list.lu (C.V.); jerome.guillot@list.lu (J.G.); biancarita.pistillo@list.lu (B.R.P.); emanuele.barborini@list.lu (E.B.)

[2] 3D-Oxides, F-01630 Saint Genis Pouilly, France; estelle.wagner@3d-oxides.com (E.W.); william.maudez@3d-oxides.com (W.M.); giacomo.benvenuti@3d-oxides.com (G.B.)

* Correspondence: corrado.garlisi@list.lu

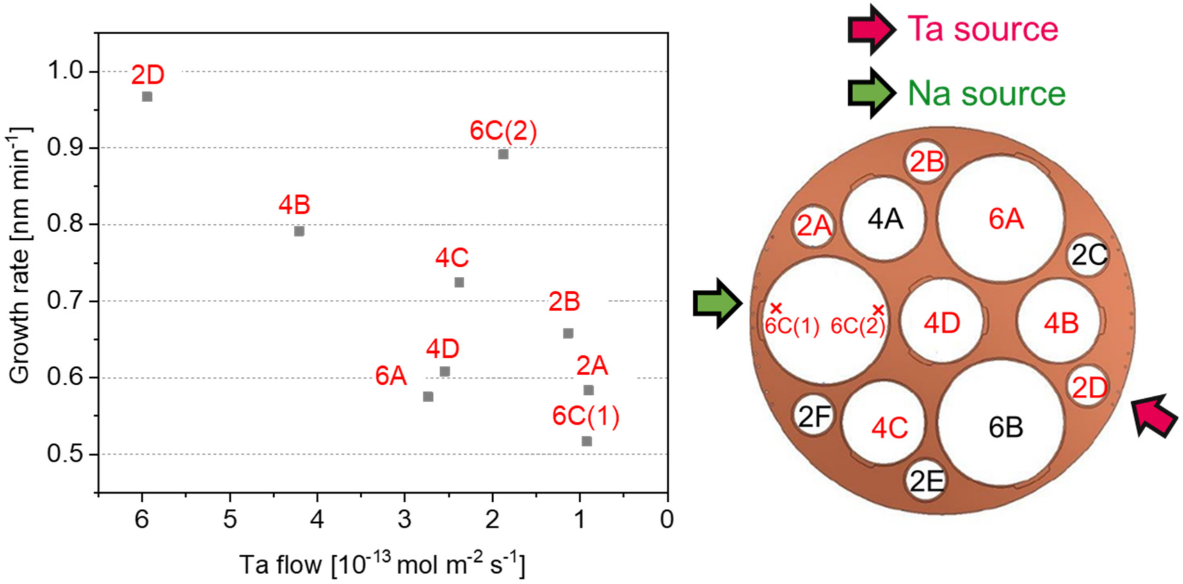

Figure S1. Measured average growth rate as function of the Ta flow calculated for each position of the samples for the 1-1 deposition batch. On the right, the schematic of the sample holder with the corresponding positions in red. The samples 6C(1) and 6C(2) come from the left edge and right edge, respectively, of the wafer 6C. The other sample come from the centre of the respective wafers.

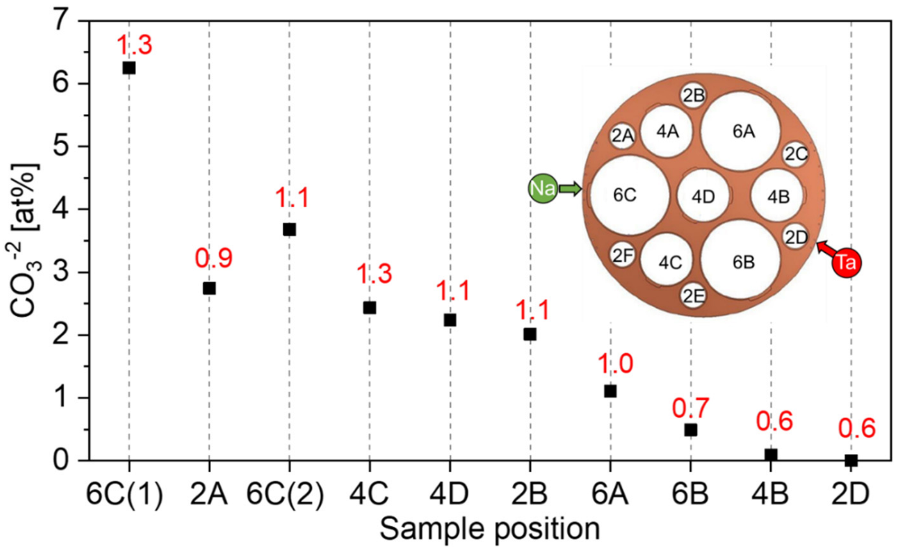

Figure S2. Carbonate amount versus sample position for the batch 1-1. The Na/Ta ratio of each sample is indicated in red above each dot. The schematic of the 1-1-configuration is displayed in the inset.

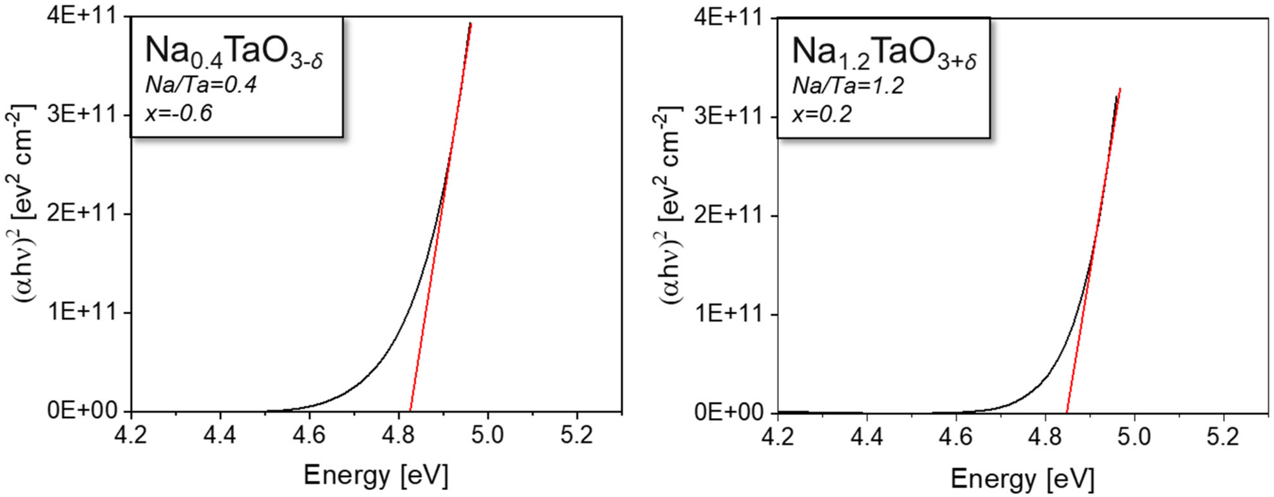

Figure S3. Tauc plots of the samples $Na_{0.4}TaO_{3-\delta}$ and $Na_{1.2}TaO_{3+\delta}$ deposited on quartz in position 2A from batch D_1-6 and B_1-6, respectively.

Table S1. Average thickness with the corresponding standard deviation. The deposition time is 120 min for the batch 1-1 and 30 min for the batches prepared in 1-6 configuration, respectively. The standard deviation refers to measurements taken in different points of the same wafer.

Sample position	Deposition batch									
	1-1		A_1-6		B_1-6		C_1-6		D_1-6	
	Average thickness (nm)	Standard deviation (nm)	Average thickness (nm)	Standard deviation (nm)	Average thickness (nm)	Standard deviation (nm)	Average thickness (nm)	Standard deviation (nm)	Average thickness (nm)	Standard deviation (nm)
6C	77	26	111	19	230	66	212	44	212	83
2A	70	3	165	1	230	13	208	5	192	15
4C	87	16	139	16	192	49	150	11	144	15
4D	73	10	139	19	137	29	125	35	114	21
2B	79	4	131	11	125	19	104	9	94	5
6A	69	6	104	16	92	11	84	10	74	20
2D	116	3	108	18	82	3	61	5	57	4
4B	95	14	100	12	89	8	71	13	64	10

Table S2. Stoichiometry, Na/Ta and *x* of samples deposited in the five deposition batches.

Sample position	Deposition batch														
	1-1			A_1-6			B_1-6			C_1-6			D_1-6		
	Stoichiometry[1]	Na/Ta	*x*	Stoichiometry[1]	Na/Ta	*x*	Stoichiometry[1]	Na/Ta	*x*	Stoichiometry[1]	Na/Ta	*x*	Stoichiometry[1]	Na/Ta	*x*
6C(1)[2]	$Na_{1.3}TaO_{3+\delta}$	1.3	0.3	$Na_{0.8}TaO_{3-\delta}$	0.8	−0.2	$Na_{1.5}TaO_{3+\delta}$	1.5	0.5	$Na_{0.6}TaO_{3-\delta}$	0.6	−0.4	$Na_{0.5}TaO_{3-\delta}$	0.5	−0.5
2A	$Na_{0.9}TaO_{3.0}$	0.9	−0.1	$Na_{0.5}TaO_{3-\delta}$	0.5	−0.5	$Na_{1.2}TaO_{3+\delta}$	1.2	0.2	$Na_{0.5}TaO_{3-\delta}$	0.5	−0.5	$Na_{0.4}TaO_{3-\delta}$	0.4	−0.6
6C(2)[3]	$Na_{1.1}TaO_{3+\delta}$	1.1	0.1	$Na_{0.6}TaO_{3-\delta}$	0.6	−0.4	$Na_{1.3}TaO_{3+\delta}$	1.3	0.3	$Na_{0.6}TaO_{3-\delta}$	0.6	−0.4	$Na_{0.4}TaO_{3-\delta}$	0.4	−0.6
4C	$Na_{1.3}TaO_{3+\delta}$	1.3	0.3	$Na_{0.7}TaO_{3-\delta}$	0.7	−0.3	$Na_{1.2}TaO_{3+\delta}$	1.2	0.2	$Na_{0.7}TaO_{3-\delta}$	0.7	−0.3	$Na_{0.5}TaO_{3-\delta}$	0.5	−0.5
4D	$Na_{1.1}TaO_{3+\delta}$	1.1	0.1	$Na_{0.7}TaO_{3-\delta}$	0.7	−0.3	$Na_{1.5}TaO_{3+\delta}$	1.5	0.5	$Na_{0.7}TaO_{3-\delta}$	0.7	−0.3	$Na_{0.5}TaO_{3-\delta}$	0.5	−0.5
2B	$Na_{1.1}TaO_{3+\delta}$	1.1	0.1	$Na_{0.6}TaO_{3-\delta}$	0.6	−0.4	$Na_{1.2}TaO_{3+\delta}$	1.2	0.2	$Na_{0.6}TaO_{3-\delta}$	0.6	−0.4	$Na_{0.5}TaO_{3-\delta}$	0.5	−0.5
6A	$Na_{1.0}TaO_{3.0}$	1.0	0.0	$Na_{1.0}TaO_{3-\delta}$	1.0	0.0	$Na_{1.5}TaO_{3+\delta}$	1.5	0.5	$Na_{0.7}TaO_{3-\delta}$	0.7	−0.3	$Na_{0.8}TaO_{3-\delta}$	0.8	−0.2
2D	$Na_{0.6}TaO_{3-\delta}$	0.6	−0.4	$Na_{1.1}TaO_{3-\delta}$	1.1	0.1	$Na_{1.2}TaO_{3+\delta}$	1.2	0.2	$Na_{0.9}TaO_{3-\delta}$	0.9	−0.1	$Na_{0.8}TaO_{3-\delta}$	0.8	−0.2
4B	$Na_{0.6}TaO_{3-\delta}$	0.6	−0.4	$Na_{1.0}TaO_{3-\delta}$	1.0	0.0	$Na_{1.3}TaO_{3+\delta}$	1.3	0.3	$Na_{1.0}TaO_{3.0}$	1.0	0.0;	$Na_{0.8}TaO_{3-\delta}$	0.8	−0.2

[1] The stoichiometry refers to 3 cm x 3 cm samples carved from center of the wafer except for 6C(1) and 6C(2);

[2] Sample carved from the left edge of the 6C wafer as shown in Figure 2;

[3] Sample carved from the right edge of the 6C wafer as shown in Figure 2.
